# Regulation of RhoA Activity by Adhesion Molecules and Mechanotransduction

**DOI:** 10.2174/1566524014666140128104541

**Published:** 2014-02

**Authors:** R.J. Marjoram, E.C. Lessey, K. Burridge

**Affiliations:** Department of Cell Biology and Physiology, Lineberger Comprehensive Cancer Center, University of North Carolina, Chapel Hill, NC 27599, USA

**Keywords:** Actin, adhesion, cytoskeleton, GTPase, force, mechanotransduction, RhoA.

## Abstract

The low molecular weight GTP-binding protein RhoA regulates many cellular events, including cell migration, organization of the cytoskeleton, cell adhesion, progress through the cell cycle and gene expression. Physical forces influence these cellular processes in part by regulating RhoA activity through mechanotransduction of cell adhesion molecules (e.g. integrins, cadherins, Ig superfamily molecules). RhoA activity is regulated by guanine nucleotide exchange factors (GEFs) and GTPase activating proteins (GAPs) that are themselves regulated by many different signaling pathways. Significantly, the engagement of many cell adhesion molecules can affect RhoA activity in both positive and negative ways. In this brief review, we consider how RhoA activity is regulated downstream from cell adhesion molecules and mechanical force. Finally, we highlight the importance of mechanotransduction signaling to RhoA in normal cell biology as well as in certain pathological states.

## INTRODUCTION

Two cell types exist: those with rigid cell walls (plants and most bacteria) and those lacking cell walls (animal cells). All cells experience mechanical forces, either imposed on them from the external environment or generated within them by their force-generating cytoskeletal elements. This brief review concerns the response of animal cells to mechanical forces. In the absence of a cell wall animal cells employ dynamic architectures in order to adjust to mechanical cues [1, 2]. In response to external forces cells may stiffen, change their shape or alter their behavior including gene expression. These changes involve multiple signaling pathways (ion channels, Rho GTPases, tyrosine and serine kinases, etc.), and many of the responses ultimately affect the cytoskeleton. The translation of mechanical forces by cells has an impact on multiple aspects of biology from embryogenesis to pathogenesis.

Cell adhesion is a crucial function in metazoans and provides organization, structure, communication, and cohesion within complex multicellular organisms [3]. Genes encoding proteins important for cell-cell or cell-matrix adhesion are highly conserved throughout evolution [3]. Cell adhesion to the surrounding microenvironment is a primary conduit for force transfer to and from the cell. There are two interfaces of cellular adhesion: cell-cell and cell-extracellular matrix (ECM) adhesion. Each interface has unique components but both link directly to the cell’s actin cytoskeleton [4]. Integrins are cell adhesion molecules that serve as the primary linkage of cells to components of the ECM (e.g. collagen, fibronectin, and laminin), but some integrins can also have a role in cell-cell adhesion [5]. A common interface of cell-cell adhesion is the adherens junction, and cadherins are the primary adhesion molecules involved in these cellular structures [6]. A major signaling node for both interfaces is RhoA, a member of the Rho family of small GTPases. Importantly, when mechanical forces are applied to a variety of cell adhesion molecules, RhoA signaling is triggered [7].

How mechanical force is transmitted to cells and affects cell signaling pathways has generated considerable interest for some time, and significant progress has been achieved. However, much still remains to be learned. In this review, we highlight some of the recent findings involving RhoA GTPase signaling initiated by mechanotransduction through cell adhesion molecules and place it in the context of normal biology and disease.

## MECHANOTRANSDUCTION

**Fig. (1):**
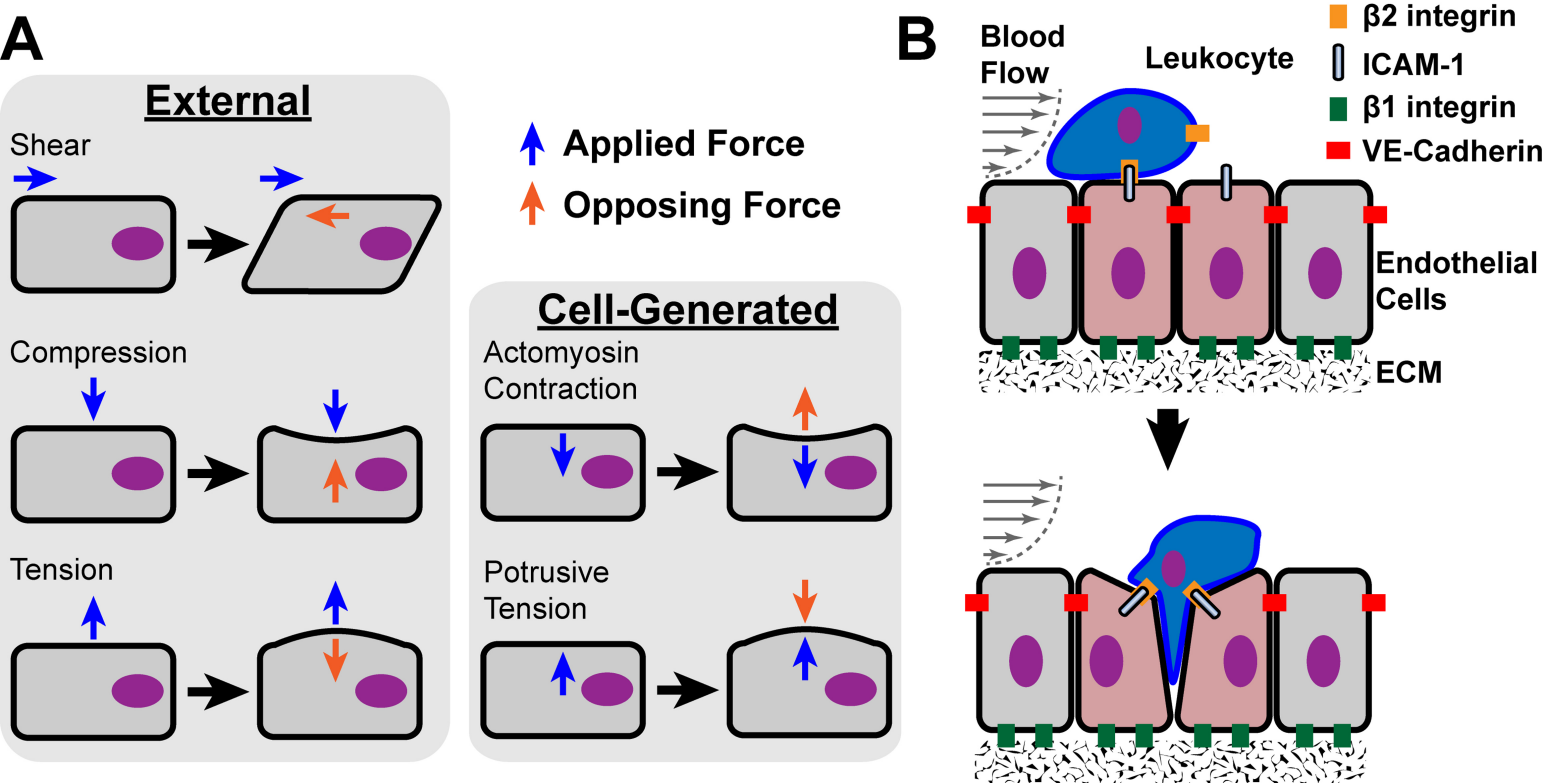
Mechanical forces in cell physiology. *A*, Diagrams of external and cell-generated mechanical forces that are applied to cells. Blue arrows depict the types of applied mechanical force vectors on cell bodies. Orange arrows represent the balancing opposing force vectors that coincide with the morphological response of the cell body. *B*, Transendothelial migration of leukocytes through endothelial cell (EC) junctions at sites of inflammation (red tinted cells) provides a model that likely incorporates all of the types of external and cell-generated forces and various adhesion receptors. Shear stresses are applied to both ECs and the adherent leukocyte due to the flow of blood. The adhesive interactions illustrated during leukocyte transendothelial migration (i.e. EC-EC, EC-leukocyte, and EC-ECM interactions) likely involve combinations of external and cell-generated forces applied through the various cell adhesion molecules: β2- and β1-integrins, ICAM-1, and VE-cadherin. The mechanotransduction by these adhesion receptors contributes to the passage of leukocytes through the endothelium without disrupting this layer.

The term mechanotransduction is defined as the ability of a cell to convert an applied physical force into a biochemical signal that induces a cellular response [4]. There are two origins for the forces that cells experience: arising from either external or intracellular sources (Fig. **1A**). External forces are applied to cells in the form of shear, compressive, and tensile stresses originating from the extracellular environment. Intracellular forces originate either from osmotic pressure or from the cytoskeletal systems where force can be generated either by polymerization of cytoskeletal structures such as actin filaments or through the action of motor proteins (e.g. myosins acting on actin filaments, or kinesins and dyneins acting on microtubules) [7]. The ability of cells to have quick and concerted responses to externally applied forces is based on the fact that the cellular components are under isometric tension [4]. The fundamental load bearing structure is the cytoskeleton, and it is composed of three types of polymeric fibers: filamentous actin (F-actin), intermediate filaments (e.g. keratin, vimentin, and lamin), and microtubules (tubulin) [8]. The integration of the cytoskeleton, adhesion receptors, and certain signaling factors form the cell’s force sensing machinery.

## FORCE-SENSING IN NORMAL CELL BIOLOGY AND PATHOLOGY

Mechanotransduction is involved in cellular responses from bacteria to humans and influences many aspects of normal cell biology but also contributes to many pathologies [9]. In tissues of vertebrates, external and cell-generated forces are important in diverse cellular activities ranging from cell migration to morphogenesis. Cell migration is important in embryonic development, immune system surveillance, and wound healing. Cells can migrate individually (e.g. leukocytes) or as interconnected collective groups (e.g. *Drosophila* border cells) [10-12]. Locomotion of cells requires generation of forces within the cell that are applied externally through cell adhesion molecules to the extracellular environment [13]. Cell migration is rarely if ever random within an organism, but is directed by environmental cues, such as gradients of soluble factors (chemotaxis), gradients of ECM components (haptotaxis) or gradients of physical stiffness (durotaxis) [10]. Directed cell migration requires the cell to polarize through integration of intracellular signaling pathways (e.g. Rho GTPase signaling) and extracellular stimuli [10, 14, 15]. Transendothelial migration of leukocytes during inflammation is a classic example of directed single cell locomotion during which cell-generated and external forces are applied to and from the leukocyte (Fig. **1B**). Leukocyte adhesion and migration through endothelial cells (ECs) at the site of inflammation occurs under conditions of shear stress produced by blood flow. Cell-generated forces are produced within both the leukocyte and ECs during adhesive and migratory interactions and are dynamically regulated through each cell’s force sensing machinery.

Tissue and organ morphogenesis rely heavily on collective cell migration during development, but this collective response also requires mechanosensing of transmitted stresses through cell-cell adhesions [16]. Cadherin linkages are crucial for collective migration, maintenance of cell polarity, and tissue morphogenesis [17-19]. Adherens junctions and ECM contacts with their connections to the actomyosin network are important mediators of tissue elongation during oscillating myosin contractions in *Drosophila* embryos [20-22]. Actomyosin contractility was also shown to be important in Zebrafish epithelial cell spreading during embryo gastrulation [23]. Epithelial cell polarization and remodeling during organ morphogenesis are also dependent on mechanosensory properties [24]. The important roles of adhesion receptors and actomyosin networks in organogenesis implicate RhoA as a regulator in these developmental processes. Several studies have shown that Rho GTPases have important roles in embryo morphogenesis [21, 25].

Even after development of an organism, physical forces on cells have significant influences on physiology. Examples include the musculoskeletal system in movement and stability, lung expansion and contraction during respiration, and the cardiovascular system during blood circulation. Considerable focus has been placed on vascular stresses and blood rheology since they have significant impacts on cellular and molecular events during both normal and pathological conditions [26]. Endothelial cells that line the vasculature directly interface with the blood and endure most of the stresses produced by its circulation.

The fluid dynamics of liquid blood are defined as non-Newtonian, where the liquid viscosity is dependent on the shear rates between adjacent fluid layers in a laminar flow system due in part to the different cell types that create a complex viscosity profile [26]. The laminar flow of blood through an artery produces a flow profile having the maximum flow velocity at the center of the vessel that decreases to the slowest velocity at the wall. The difference in flow velocities between parallel fluid layers produces a shear stress (the force per unit area between adjacent fluid layers) and a shear rate (the relative change in velocity between adjacent fluid layers) both of which are highest at the blood/artery wall boundary [26]. Shear rates at the arterial wall are in the range of 1,000 to 10,000 s^-1^ [27]. The pulsatile nature of blood flow also produces an oscillating outward radial pressure and cyclic stretching on the arterial walls. Under normal conditions, endothelial cells of arteries experience these different types of mechanical forces (i.e. shear and cyclic stress) that modulate their cellular functions some of which are controlled through RhoA signaling pathways.

Pathologies develop when normal biological processes malfunction. Both cancer and atherosclerosis are examples of pathological conditions where the physical properties and the tensional homeostasis of the afflicted tissues are altered. Atherosclerosis illustrates how mechanical forces can impact a pathological situation. Not only do regions of turbulent flow promote inflammation of the arterial wall thereby leading to the development of atherosclerotic plaques, but these structures become relatively rigid due to the deposition of excess ECM as well as due to their calcification [28]. Increased vascular stiffening promotes RhoA-dependent endothelial cell permeability to increase leukocyte transmigration [29]. Monocytes are recruited to the inflamed arterial wall where they infiltrate the tissue and differentiate into macrophages that take up large quantities of lipid to become foam cells [30]. In turn, these cells secrete growth factors that stimulate the local proliferation of vascular smooth muscle cells and promote their invasion into the intimal layer of the arterial wall. The increased rigidity of the atherosclerotic plaque likely affects signaling within the smooth muscle cells, elevating RhoA activity. This may stimulate proliferation, but it will also increase contraction, thereby causing a positive feedback cycle in which the lumen of the artery is narrowed resulting in increased turbulent flow and more inflammation of the arterial wall. Arterial wall shear rates can be significantly higher at sites of pathological stenosis caused by atherosclerotic plaques (shear rates > 10,000 s^-1^) [27]. Stenosis of arteries also produces turbulent flow that diverges from laminar flow, producing zones of flow deceleration and acceleration, streamline separation, and flow vortices [26]. These physical changes at sites of atherosclerotic plaques all contribute to the progression of the pathology.

In cancer, the abnormal tissue within the tumor typically becomes firmer than the surrounding normal tissue, and this change in cellular environment promotes tumor progression [31-33]. Notably, cancers are often first detected by palpation of the harder tumor tissue. The increased stiffness is attributed to unregulated tumor cell proliferation and increased deposition of the ECM [34]. Paszek *et al.* showed that increasing ECM stiffness promoted oncogenesis through an integrin/RhoA mechanosensory mechanism [34]. Tumor stiffness also can promote cancer cell migration and metastasis, processes involving RhoA signaling [33, 35-37].

### Rho GTPases AND RhoA

**Fig. (2):**
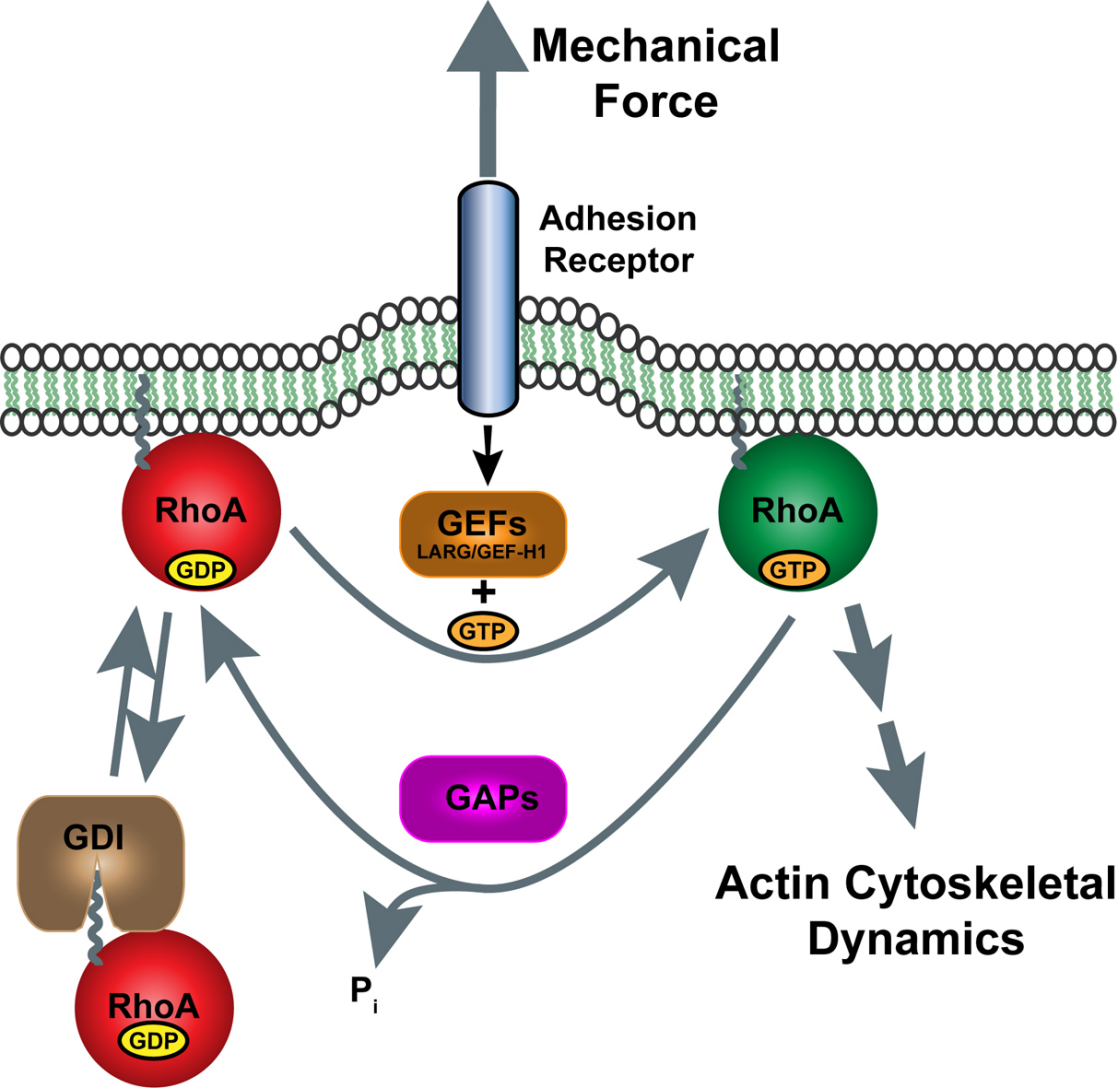
Regulation of RhoA activity by mechanical force on adhesion receptors. Tensional force applied to cell adhesion receptors (e.g. integrins or cadherins) is transduced through the plasma membrane to activate certain Rho GEFs (e.g. LARG and GEF-H1) that promote exchange of GDP with GTP on RhoA. GTP-bound RhoA stimulates downstream effectors (e.g. ROCK and mDia) that regulate actin cytoskeletal dynamics. Rho GAPs shut off RhoA signaling through hydrolysis of GTP to restore inactive GDP-bound RhoA. RhoA signaling is further attenuated by binding of GDIs to RhoA, extraction from the membrane and sequestering in the cytosol.

Rho GTPases are a subgroup of the Ras superfamily of small guanine nucleotide-binding proteins and they regulate many cellular activities including cytoskeletal dynamics, cell adhesion, vesicle trafficking, progress through the cell cycle and gene expression [38]. These small GTPases function as molecular switches that cycle between a GTP-bound, active conformation and a GDP-bound, inactive conformation (Fig. **2**) [39]. This cycling between bound GDP or GTP (i.e. their activity) is regulated by three types of cytoplasmic factors, Guanine nucleotide Exchange Factors (GEFs), GTPase activating proteins (GAPs), and Guanine nucleotide dissociation inhibitors (GDIs). GEFs promote the exchange of the bound nucleotide and because the cytosolic ratio of GTP to GDP is approximately 10:1, the exchange favors the generation of predominantly the GTP-bound form of Rho protein. GAPs stimulate the GTPase activity of Rho proteins, thereby converting the GTP to GDP and blocking the interaction of the Rho protein with its effectors. GDIs extract membrane-bound GTPases into the cytosol, where they are sequestered and prevented from being activated or interacting with effectors [40]. Of the approximately 20 mammalian members of the Rho GTPase family, RhoA, Rac1, and CDC42 are the best characterized, and their activities typically control the formation of distinct actin cytoskeletal structures: stress fibers, lamellipodia, and filopodia, respectively [41].

RhoA has many effectors, several of which influence the actin cytoskeleton. Initially, it was discovered that active RhoA stimulates myosin activity and actomyosin contractility by elevating the phosphorylation of the regulatory myosin light chain [42]. This was shown to be mediated by two pathways, direct phosphorylation of the light chain by the RhoA effector, Rho kinase (ROCK) [43] and by this same kinase phosphorylating and inhibiting the myosin light chain phosphatase [44]. Activated RhoA stimulates actin polymerization by binding to and stimulating the formin mDia [45]. RhoA activation of ROCK also stabilizes F-actin by activating LIM kinase, which phosphorylates and inhibits the F-actin severing protein, cofilin [46]. With these important connections to the load bearing cellular architecture, RhoA has been a main focal point in studies of cellular response to mechanical forces. Indeed, mechanosensing of both external and cell-generated forces appear to be transduced through shared signaling pathways that impact RhoA activity [47]. These studies point to RhoA as an important node in mechanotransduction.

## RhoA REGULATION BY CELL ADHESION MOLECULES AND MECHANOTRANSDUCTION

Cell adhesion molecules provide the linkages that transduce mechanical stimuli back and forth between the intracellular and extracellular compartments. Both vertebrate and invertebrate cells have adhesion receptors, but most of the research on mechanotransduction has been done on vertebrate molecules. In more detail below, we focus on vertebrate proteins, including integrins, cadherins, and other cell adhesion molecules, which are important research targets in the field of mechanotransduction. Interestingly, a recent *in vivo* study on *Drosophila* myotendinous juctions showed integrin turnover was regulated by force [48]. For more information on mechanical forces on invertebrate cells during development we direct the reader to some informative reviews on this subject [49, 50].

### Integrins

Integrins are a family of proteins that function as adhesion receptors on the surface of cells and are heterodimers composed of eighteen α subunits and eight β subunits to form 24 distinct integrins [5, 51]. The 24 integrins can be further subgrouped into RGD-binding, collagen-binding, laminin-binding, or leukocyte-specific receptors. In general, integrins are bidirectional signaling receptors (inside-out and outside-in signaling mechanisms) that connect the ECM to the actin cytoskeleton of cells [52]. They transition between several conformational states, an inactive conformation (low affinity) to activated conformations of intermediate or high affinities upon ligation or through intracellular signals [5, 53]. Integrins cluster at focal adhesions where they couple to bundles of actin filaments (stress fibers) and provide attachment to the ECM [54].

Since the initial characterization of integrins, these surface receptors have been recognized as transmembrane links between the external ECM and the internal actin cytoskeleton [55]. It has been intuitive that a protein designed to withstand mechanical forces should also serve as a transducer of these applied forces. A study by Friedland *et al.* reported that α5β1 integrin compensates for increased force on α5β1 by a “catch bond” mechanism, which increases the bond strength of α5β1 for fibronectin, as well as stimulating Focal Adhesion Kinase (FAK) signaling [56]. Structural analysis and molecular dynamics of αIIbβ3 integrin revealed that force application by the actin cytoskeleton induced the high affinity conformation of αIIbβ3 and reverted the affinity upon actin disassembly [57]. Also, studies on cell tractional forces on the ECM through integrins support the role of integrins as part of the cell’s mechanosensory equipment [58-64]. These data show how integrins are an integral part of the load bearing machinery of cells and suggest that they can transduce mechanical stimuli into biochemical signals.

When the first assay to measure RhoA activity was being developed it was applied to examine how this activity was affected by cell adhesion to fibronectin [65]. Somewhat unexpectedly, it was observed that RhoA activity initially decreased for the first 10-20 minutes following adhesion but then increased above the baseline over the next hour. Pursuing the mechanism for the decrease in RhoA activity, a pathway was identified by which integrin engagement activated the tyrosine kinase Src that phosphorylated and activated the RhoA GAP p190RhoGAP thereby lowering RhoA activity [66]. Additional kinases have been implicated in some situations [67]. It was argued that the initial depression in RhoA activity facilitated cell spreading by preventing excessive contractility, and evidence in support of this idea was presented [68]. The subsequent activation of RhoA correlates with the time course of focal adhesion assembly and the development of increased traction on the underlying matrix as spreading cells polarize and begin to migrate. Several GEFs have been identified being activated during adhesion to fibronectin, including Lsc/p115RhoGEF and LARG [69], and p190RhoGEF [70], with this latter GEF being regulated downstream of the tyrosine kinases FAK and Pyk2 [71]. These studies support a role for RhoA in adhesion receptor signaling and mechanosensing.

As integrins are major links between the ECM and the actin cytoskeleton, it was anticipated that they would be involved in mechanotransduction. The first evidence for this was provided when magnetic beads coated with fibronectin were applied to cells and then exposed to a twisting force. In response to the force, stiffening of the cell cortex was detected [72]. A similar stiffening or reinforcement was observed when fibronectin-coated beads adhering to cells were manipulated using laser tweezers [73]. Applying short pulses of force to similar beads using magnetic tweezers revealed that active RhoA was required for the stiffening response of endothelial cells [74]. A study by Féral *et al.* found CD98hc, a membrane protein on fibroblasts, is necessary for extracellular fibronectin matrix assembly through association with integrins [75]. They further showed that CD98hc promotes integrin-mediated activation of RhoA and actomyosin contractility to produce traction forces on the ECM through the integrins. Another report by this group showed that CD98hc is important in skin homeostasis because CD98hc in keratinocytes promotes integrin activation of RhoA leading to cell proliferation and migration [76].

Exploring which GEFs might be involved in integrin-mediated activation of RhoA downstream of mechanical force applied to β1 integrins on fibroblasts, Guilluy *et al.* identified both LARG and GEF-H1, although, surprisingly, the pathways leading to their activation were distinct [77]. LARG was activated downstream of the Src family kinase Fyn, whereas GEF-H1 activation involved the Ras pathway downstream of the focal adhesion kinase (FAK) with phosphorylation of GEF-H1 by ERK [77]. These studies support the role of RhoA in cellular responses and point to LARG and GEF-H1 as regulating RhoA activity during integrin-mediated mechanotransduction.

### Cadherins

Cadherins are a family of Ca^2+^-dependent, primarily homophilic adhesion receptors found on most cells that mediate cell-cell binding interactions. In epithelial tissues cadherins are concentrated within adherens junctions, which in most mature tissues are relatively stable structures. However, during development when these adhesions are assembling they reveal dynamic linkages between cadherins and cytoskeletal networks [78]. Additionally, the adherens junctions of endothelial cells need to be dynamic during inflammation, when endothelial junctions transiently open to allow passage of leukocytes from the blood into tissues. It is known that ROCK and Dia downstream of RhoA have divergent effects on the stability of adherens junctions; ROCK activity destabilizes junctions whereas Dia activity strengthens them [79]. A recent study by Ngok *et al.* determined that Syx (a Rho GEF, also known as PLEKHG5 and KIAA0720) regulates downstream activation of ROCK or Dia depending on its junctional localization controlled by phosphorylation of Syx at S806 [80]. During leukocyte transendothelial migration, VE-cadherin, the cadherin expressed in endothelial cells, is seen to be lost from the sites of leukocyte passage [81], although how this occurs remains controversial [82]. The most popular model for studying cadherins and the dynamics of adherens junctions has been to experimentally remove Ca^2+^ from the extracellular medium of cells in culture. Loss of extracellular Ca^2+^ causes adherens junction disassembly and initially this was interpreted as simply due to breaking the homophilic interactions between cadherin extracellular domains on opposing cells. However, Citi elegantly demonstrated that the disassembly of adherens junctions under these conditions was blocked by certain inhibitors of protein kinase signaling, implying a more complex situation [83]. Subsequent work showed that the kinase inhibitor H-7 was preventing junction disassembly by inhibiting cell contractility [84]. Later, H-7 was shown to be a potent inhibitor of ROCK [85]. Together these results implied that adherens junctions are under tension from their associated actin filaments and that this tension is critical in the disassembly of the junctions when cadherin adhesion is weakened by removal of Ca^2+^. Many other lines of evidence point to tension contributing not only to the disassembly of cadherin-based adhesions but also to their assembly [86]. Recent work demonstrated that α-catenin, a linker protein associated with cadherins, changes its conformation in response to tension on epithelial junctions to expose a cryptic site that can bind vinculin [87]. In parallel, it was shown that vinculin is recruited to adherens junctions in response to mechanical tension and that tension on E-cadherin leads to a stiffening response that is dependent on vinculin [88]. Interestingly, work by Borghi *et al.* showed that the cytoplasmic tail of E-cadherin is under mechanical tension, both when it is in adherens junctions and when it is free in the plasma membrane away from the junctions [89].

Cadherin engagement was found to depress RhoA activity [90, 91] and, as for integrin-mediated adhesion, activation of p190RhoGAP was implicated in this initial decrease in RhoA activity [92]. However, more recent work identified myosin IXa as a critical GAP depressing RhoA activity at newly developing adherens junctions [93]. Other studies have observed that engagement of cadherins is associated with increased RhoA activity. For example, RhoA activity increased over a prolonged time course during which keratinocytes were allowed to reform their adherens junctions after calcium was restored to the medium [94]. Similarly, prolonged engagement of VE-cadherin was also found to be associated with elevated RhoA activity and this was shown to depend on mechanical force being exerted on the adhesions [95]. The finding that force on VE-cadherin stimulates RhoA activity has been directly confirmed using magnetic beads coated with the extracellular domain of VE-cadherin (Marjoram, unpublished results). The contribution of force on cadherins versus simple cadherin engagement may explain some of the apparent discrepancies between these studies with respect to whether cadherin-mediated adhesion inhibits or activates RhoA. However, different cadherins may also differ in their signaling to RhoA, as suggested by the finding that engagement of N-Cadherin in cultures of myoblasts was associated with increased RhoA activity rather than decreased activity [96].

### Ig Superfamily Adhesion Molecules

The largest family of adhesion molecules are those with immunoglobulin-like domains. These are highly heterogeneous both in regard to their binding partners and with respect to their cytoplasmic domains. Here we will briefly discuss a few examples, PECAM-1, ICAM-1, JAM-A, and Nectin.

### PECAM-1

Platelet Endothelial Cell Adhesion Molecule-1 (PECAM-1; also known as CD31) is a type-1 membrane protein belonging to the immunoglobulin superfamily that is expressed on certain blood cells and endothelial cells [97]. On these cell types, PECAM-1 primarily acts as a cell-cell adhesion molecule through homophilic interactions with PECAM-1 molecules on contacting cells, although some heterophilic interactions have been reported (e.g. CD177) [98]. PECAM-1 was initially found to have an important role during inflammation and specifically in the process of leukocyte transendothelial migration [98]. Leukocyte adhesion to the vascular wall through attachment to endothelial cells partially occurs through PECAM-1 associations between these two cell types [99, 100]. PECAM-1 is also important in forming linkages within adherens junctions of vascular endothelial cells [101, 102]. These findings suggested that PECAM-1 might serve as a transducer of mechanical forces within the blood and vascular cells where it is expressed.

Early studies by Osawa *et al.* reported that mechanical force on PECAM-1 of endothelial cells stimulated tyrosine phosphorylation of the cytoplasmic domain of PECAM-1, and induced its association with the signaling proteins SHP2 and Gab1, and promoted ERK activation [103, 104]. Tzima *et al*. showed that PECAM-1 is a component of a mechanosensory complex along with Vascular Endothelial cell cadherin (VE-cadherin) and Vascular Endothelial Growth Factor Receptor-2 (VEGFR-2) in vascular endothelial cells that modulates cellular responses to fluid flow and shear stress [105]. Another study identified cytoskeletal-linked Fyn tyrosine kinase as important in PECAM-1-mediated mechanotransduction in endothelial cells [106]. These studies support PECAM-1 as a transducer of mechanical force to cellular biochemical signals within endothelial cells.

A recent study by Collins *et al.* linked PECAM-1 mechanotransduction in endothelial cells to activation of RhoA [107]. These investigators showed that direct application of tension on PECAM activates RhoA, but interestingly this depends on the integrin by which the cells are adhering to the matrix and is mediated by LARG and GEF-H1 [107]. The PECAM and integrins were not closely associated but largely distributed on opposite surfaces of the endothelial cells suggesting that the mechanical tension on PECAM was being transmitted to the integrins *via *cytoskeletal connections. The Rho GEFs, LARG and GEF-H1, were identified as the effectors responsible for activating RhoA downstream of PECAM-1 mechanotransduction. Given that RhoA activation leads to cell stiffening, these authors suggested that the mechanotransduction pathway from PECAM-1 to RhoA may contribute to the varying vessel stiffness observed under different hemodynamic forces. Another recent study utilized tension-sensitive FRET biosensors for PECAM-1 and VE-cadherin and showed that under shear stress conditions endothelial cells relax tension on VE-cadherin and increase tension on PECAM-1 through interaction with vimentin [108]. It will be interesting to investigate this further and to determine whether other surface proteins that link to the cytoskeleton can similarly signal and induce RhoA activation by transmission of force to integrins anchoring the cell to the ECM.

### ICAM-1

Intercellular adhesion molecule-1 (ICAM-1) is an Ig domain-containing cell adhesion molecule that functions in the recruitment of leukocytes from the blood to sites of inflammation [109]. In response to inflammatory cytokines, such as interleukin-1 or tumor necrosis factor-alpha, ICAM-1 expression is greatly increased on the surface of endothelial cells and provides a strong adhesive ligand through which leukocytes adhere to the endothelial surface [110]. Initial adhesion is usually mediated by selectins, but the interaction of leukocyte integrins with ICAM-1 allows leukocytes to spread and migrate over the surface, and typically, the leukocytes migrate toward cell-cell junctions through which they migrate [111]. The clustering of ICAM-1 by leukocyte integrins triggers multiple signaling pathways that contribute to the passage of the leukocytes across the endothelium [110, 112]. Several stages in the passage of leukocytes across the endothelium involve mechanical forces, starting with tractional forces generated as the leukocytes migrate. The tractional forces exerted by locomoting leukocytes have been investigated on artificial substrata coated with ECM proteins and the forces have been found to be typically two orders of magnitude less than more slowly migrating cells such as fibroblasts [58, 113, 114]. As they invade into junctions the leukocytes generate forces that are transmitted both to the endothelial cells as well as to the underlying substratum [115, 116]. We have been interested in whether mechanical forces applied on proteins such as ICAM-1 augment signals generated by clustering or even initiate novel signaling pathways. Our preliminary results indicate that applying force to ICAM-1 amplifies the activation of RhoA that occurs downstream from engagement and clustering of ICAM-1 and that this results in a stiffening of the endothelial cell (Lessey and Burridge, unpublished results). We speculate that this stiffening response may provide a surface that is more favorable for leukocyte migration to the endothelial junctions.

### Other Members of the Ig Superfamily

There are many members of the Ig superfamily that function as cell surface adhesion receptors, although little is known if they function as force sensors and activators of RhoA. Two family members, junctional adhesion molecule-A (JAM-A) and Nectin-1, are promising candidates for transducing mechanical forces. JAM-A is a homophilic adhesion receptor present at tight junctions of epithelial and endothelial cells as well as on surfaces of some blood cells and functions in barrier function and cell migration [117, 118]. RhoA signaling was shown to be important for the relocalization of JAM-A on the surface of epithelial cells [119] and brain endothelial cells [120] during inflammation. Another group showed that JAM-A homophilic interactions within tight junctions of endothelial cells could be disassociated through a higher affinity interaction with LFA-1 on leukocytes, thus promoting transendothelial migration [121]. These results show JAM-A is associated with processes that involve mechanical tension and suggest it could function as a force sensor, however this speculation will need to be tested.

Nectins are Ig-like adhesion receptors that form homo- and heterophilic interactions within adherens junctions [122]. Whether or not nectins can act as force sensors is not known, but the cytoplasmic tails of nectins can bind afadin (AF6), which links the adhesion receptor to the actin cytoskeleton [123]. Activation of RhoA by nectin ligation has not been thoroughly explored, although entry of herpes simplex virus-1 into cells through a phagocytic mechanism involved nectin clustering and RhoA activation [124]. Similar to the situation with JAM-A, determining if nectins can transduce physical stresses will be important in understanding how endothelial cells respond to applied forces.

## CONCLUSION

Cells in the body have developed ways to respond to the mechanical forces to which they are constantly exposed. This process of mechanotransduction involves translation of physical forces into biochemical signaling pathways and is important in normal physiology but also contributes to the pathologies of diseases such as cancer and atherosclerosis. Cell adhesion molecules and the adhesions they make appear to be the main sites at which mechanotransduction occurs. Downstream from cell adhesion molecules, RhoA is an important signaling node that regulates many of the cytoskeletal changes induced by force. Many unanswered questions remain to be addressed concerning RhoA signaling in mechanotransduction. The GEFs LARG and GEF-H1 have been identified in the pathway activated by tension on integrins but whether these same GEFs are universally activated following application of mechanical force to different adhesion molecules has not been determined. Similarly, the role of GAPs and other RhoA regulatory components in mechano-transduction needs to be investigated. A critical question that we have not addressed in this brief review is the nature of the mechanosensor and which proteins in adhesion complexes undergo physical deformation to initiate the signaling pathways activated by mechanical force. Historically, mechanotransduction has been a difficult research area to approach. Rapid progress, however, is now being made following the development of techniques to apply force experimentally to cells, which has enabled analysis of force-activated signaling pathways. Until recently the idea that one might manipulate mechanotransduction path-ways therapeutically seemed remote. However, given that many mechanotransduction pathways converge on RhoA and that there are ways to modulate RhoA signaling, suddenly it becomes appealing to contemplate therapeutic intervention in the treatment of various disease situations in which mechanotrans-duction plays a role. The future of this area promises to be exciting.
